# Analysis of Genetic Diversity and Race Genetic Structure of Major Horse Breeds in Xinjiang, China

**DOI:** 10.3390/ani15182690

**Published:** 2025-09-14

**Authors:** Linlang Hou, Ablat Sulayman, Yaqi Zeng, Lu Zhou, Ainiwan Aimaier, Adiljan Kader, Lei Shi

**Affiliations:** 1 Xinjiang Key Laboratory for Ecological Adaptation and Evolution of Extreme Environment Organism, College of Life Sciences, Xinjiang Agricultural University, Urumqi 830052, China; houlinlang11@126.com (L.H.); zhoulu@xjau.edu.cn (L.Z.); 2 Institute of Animal Husbandry, Xinjiang Academy of Animal Science, Urumqi 830011, China; iblat2009@sina.cn; 3 Xinjiang Key Laboratory of Equine Breeding and Exercise Physiology, College of Animal Sciences, Xinjiang Agricultural University, Urumqi 830052, China; zengyaqi@xjau.edu.cn; 4 Pratacultural Research Institute, Xinjiang Academy of Animal Science, Urumqi 830011, China; ainiwanaimaier@126.com

**Keywords:** Kyrgyz horse, Barkol horse, genetic diversity, microsatellite, Chinese indigenous horse

## Abstract

The study of horse genetic diversity is imperative for informing conservation strategies, safeguarding ancestral lineages, and enhancing breed adaptability to environmental and disease pressures. This study employed 13 microsatellite markers with fluorescent-labeled capillary electrophoresis to analyze the genetic diversity of the Kyrgyz horse (*n* = 30) and Barkol horse (*n* = 30) for the first time, comparing them with three other indigenous horse breeds (*n* = 30 per breed) from Xinjiang, China. A total of 208 alleles were detected. The Polymorphic Information Content (PIC) results from GenAlEx 6.5115 show that all loci, except for the HTG06 locus in the Yanqi horse races, were highly polymorphic (PIC > 0.5), indicating a high level of genetic diversity across the five races. Among the five races, the Kyrgyz horse exhibited the lowest mean values for the effective number of alleles (Ne), observed heterozygosity (Ho), and expected heterozygosity (He), which were 6.025, 0.737, and 0.810, respectively. In contrast, the Barkol horse showed the highest mean number of alleles (Na), Ne, and He values, at 11.308, 6.330, and 0.816, respectively. Principal Coordinate Analysis (PCoA), performed using GenAlEx 6.5115, revealed the smallest genetic distance between the Kyrgyz and Yanqi horse breeds. Combined with phylogenetic tree and clustering analysis results, this supports the hypothesis that the two breeds share a common origin. This study offers valuable scientific insights for conserving and utilizing the genetic resources of indigenous Xinjiang horse breeds, specifically the Kyrgyz and Barkol horses.

## Simple Summary

This study analyzed the genetic diversity of a total of five horse breeds from Xinjiang using 13 microsatellite markers. Results indicated high genetic diversity and low inbreeding levels across all races. Principal component analysis, phylogenetic reconstruction, and Bayesian clustering consistently showed a close genetic relationship between the Kyrgyz horse and Yanqi horse, suggesting shared ancestry. These findings expand the genetic resource database for Xinjiang’s indigenous equine breeds and provide new insights into the origin of the Kyrgyz horse.

## 1. Introduction

The horse (*Equus caballus*), one of the earliest domesticated livestock species, historically served as humanity’s primary means of transportation and draft power. It played irreplaceable roles in activities such as transport, cultivation, and warfare, significantly accelerating the geographical expansion of trade and language [1]. However, the proliferation of motorized transportation and agricultural mechanization in the late 20th century precipitated substantial declines in indigenous horse races. For instance, China’s equine inventory decreased from 8.766 million in 2000 to 3.591 million in 2023 (China Statistical Yearbook, 2024 edition), heightening the risk of genetic erosion. Genetic diversity, the foundation for species adaptation to environmental change and disease challenges [2], also provides essential genetic resources for breed improvement. Consequently, the systematic assessment of genetic structure in endangered local breeds is imperative for conservation biology.

Microsatellite markers are extensively utilized in livestock genetic diversity research due to their genome-wide distribution, elevated polymorphism levels, codominant inheritance pattern, transferability across related species, and relative technical simplicity [3,4,5]. Analysis of microsatellite allelic distributions facilitates the reconstruction of evolutionary relationships among races [6]. For instance, Duderstadt et al. [7] documented high polymorphism and significant differentiation in Dülmen horses compared to 17 other European breeds. Similarly, Ling et al. [8] revealed substantial genetic diversity within Chinese indigenous horse breeds. Conversely, Zeng et al. [9] reported reduced genetic diversity in Guanzhong horses relative to other Chinese breeds, attributing this decline to high inbreeding. Wang et al. [10], using 16 microsatellites, identified moderate levels of genetic diversity, differentiation, and inbreeding in Yanqi horses. While Li et al. [11] analyzed polymorphism in Kyrgyz horses using polyacrylamide gel electrophoresis (PAGE), the present study bridges a research gap by conducting the first genetic diversity assessment of both Kyrgyz and Barkol horses employing the higher-resolution technique of fluorescently labeled capillary electrophoresis.

Xinjiang Uygur Autonomous Region hosts China’s largest horse race, with an inventory of 1.135 million head in 2023, accounting for 31.6% of the national total (China Statistical Yearbook, 2024 edition). The region’s indigenous horse breeds exhibit unique evolutionary histories and adaptive traits, necessitating systematic genetic diversity studies. The Kyrgyz horse (KEKZ), developed by Kyrgyz pastoralists under the distinct environmental conditions of the western Tianshan Mountains and Pamir Plateau, consequently exhibits strong adaptability to high-altitude environments characterized by cold and hypoxia [12], constitutes a valuable genetic resource. Prolonged isolation from exotic breeds within these habitats has preserved its high genetic purity, resulting in its inclusion in China’s National Catalog of Livestock and Poultry Genetic Resources in 2009. The Barkol horse (BLK), originating in the late Qing Dynasty (mid-19th to early 20th century), was developed by Kazakh herders through crossbreeding Kazakh horses with Mongolian and other breeds [13], and was listed in the same Catalog in 2001.

The Kazakh horse (HSK) primarily inhabits the northern foothills of the Tianshan Mountains, as well as the Altay, Yili, and Tacheng regions of Xinjiang, China, extending into Kazakhstan and Mongolia. Developed through long-term selection by Kazakh pastoralists, this indigenous breed exhibits distinct phenotypic traits shaped by the combined forces of natural and artificial selection. Characterized by robustness and sturdiness, the Kazakh horse demonstrates adaptations typical of herd-keeping environments. It possesses a relatively heavy build and dual-purpose conformation (suitable for both work and production), complemented by notable milk production [14].

The Yanqi horse (YQ), a traditional Chinese indigenous breed, originates from the central mountainous region of the Tianshan Mountains within the Bayingolin Mongol Autonomous Prefecture, Xinjiang, China. This breed possesses distinct genetic characteristics, potentially shaped by historical Mongol migrations. Its evolutionary trajectory likely incorporates genetic components derived from Mongolian, Kazakh, and Circassian horses [10].

The Yili horse (YL) originated in the Yili Kazakh Autonomous Prefecture, Xinjiang, China. Developed in the mid-20th century through crossbreeding using the Kazakh horse as the maternal foundation with Orlov Trotter, Budyonny, and Don breeds, subsequent breeding programs have integrated Russian Trotter, Thoroughbred, Warmblood, and Kustanai bloodlines to enhance performance traits [15]. Currently, the Yili horse is recognized as China’s only specialized saddle horse breed.

In certain regions of Xinjiang, where large-scale pastoralism remains a dominant livelihood, these local horse breeds serve multifaceted roles that extend beyond agricultural production. In addition to providing by-products such as mare’s milk, they continue to function as essential means of transportation for herders, facilitate the movement of goods across rugged terrain, and support tourism activities. During the travel season, these horses are often employed for recreational riding and as pack animals for carrying luggage, thereby contributing both to local economies and the preservation of traditional pastoral practices.

This study employs 13 microsatellite markers to conduct race genetic analyses of Kyrgyz horse, Barkol horse, and other Xinjiang breeds (Yanqi horse, Kazakh horse, Yili horse), assessing genetic diversity, delineating race structure, and investigating phylogenetic relationships and gene flow history to provide a scientific basis for conserving and utilizing genetic resources of Xinjiang’s local horse breeds, particularly Kyrgyz horse and Barkol horse.

## 2. Materials and Methods

### 2.1. Ethical Approval

All animal experiments were approved by the Laboratory Animal Welfare and Ethics Committee of Xinjiang Agricultural University (Animal protocol number 2024036). The care and use of experimental animals were conducted in full compliance with Chinese animal welfare laws, guidelines, and policies (GB/T 35892-2018).

### 2.2. Sample Collection

Whole-blood samples were collected from 170 horses representing five distinct domestic horse breeds distributed across the Xinjiang Uygur Autonomous Region of China (Table 1). Photographs of all horse breeds included in this study are available in the Supplementary Materials (Figure S1). Jugular venipuncture was performed using EDTA-coated tubes, and samples were promptly stored at −20 °C prior to DNA extraction.

### 2.3. DNA Extraction, PCR Amplification and Microsatellite Genotyping

Genomic DNA was isolated from blood samples using the DP304-03 DNA Extraction Kit (TIANGEN, Beijing, China). DNA integrity was verified by 1% agarose gel electrophoresis, while the purity (OD260/OD280) was quantified using a NanoDrop One spectrophotometer (Thermo Fisher Scientific, Waltham, MA, USA). In this study, 13 microsatellite markers (HTG04, ASB17, HMS06, HTG06, ASB23, HMS07, AHT04, HMS03, VHL20, HMS02, ASB02, HTG07, and UCDEQ425) co-endorsed by the Food and Agriculture Organization (FAO) and the International Society for Animal Genetics (ISAG) were employed [16]. Primer sequences are listed (Table S1). PCR amplification (Bioer TC-96, Hangzhou, China) was conducted in 25 μL reactions containing 4.9 μL of 2× UTAP PCR MasterMix (ZOMANBIO, Beijing, China), 0.5 μL each of forward and reverse primers (Sangon Biotech, Shanghai, China), 2 μL DNA template, and 16.6 μL ddH_2_O. Thermal cycling parameters included: initial denaturation (98 °C, 3 min); 33 cycles of denaturation (98 °C, 15 s), annealing (53–58.6 °C, 75 s), and extension (72 °C, 30 s), final extension (72 °C, 5 min), storage at 4 °C. PCR products underwent preliminary verification via 1% agarose gel electrophoresis followed by capillary separation using an ABI 3730xl DNA Analyzer (Applied Biosystems, Foster City, CA, USA) with Applied Biosystems Pop-7™ Polymer and Hi-Di Formamide. Fluorescent fragment detection and sizing utilized GeneMapper software version 2.6.4 (Applied Biosystems, Wakefield, RI, USA).

### 2.4. Microsatellite Statistical Analysis

Hardy–Weinberg equilibrium (HWE), No. Effective Alleles (Ne), No. Alleles (Na), Observed Heterozygosity (Ho), Expected Heterozygosity (He), number of private alleles (NPA), and Principal Coordinate Analysis (PCoA) were calculated using GENALEX 6.511 [17]. Polymorphism information content (PIC), number of alleles (K), and number of individuals per locus (N) were computed using CERVUS 3.0.7 [18]. Pairwise Fst values quantifying genetic differentiation among the five Xinjiang horse races were calculated using GENALEX 6.511 [17], Wright’s F-statistic, specifically Fst, quantifies the degree of genetic differentiation among subpopulations by comparing the total genetic diversity (H_T_) in the entire race to the average genetic diversity within individual subpopulations (HS). Analysis of Molecular Variance (AMOVA) was performed using ARLEQUIN 3.1 [19] with 1000 permutation tests. Phylogenetic tree analysis was conducted using MEGA 11.0 [20] software, employing the Neighbor-Joining method based on Nei’s genetic distance matrix. Race structure analysis was carried out using STRUCTURE 2.3.4 [21,22], with K values ranging from 2 to 6 to estimate the number of distinct races (ΔK). To determine the average estimates and standard deviations for each K value, a burn-in period of 10,000 and 100,000 Markov Chain Monte Carlo (MCMC) iterations was set, and the optimal K value and genetic homogeneity for each cluster were calculated. Graphical results, including race clustering hierarchies, mean LnP(K), LnP(K), Ln′(K), |ln″(K)|, and ΔK (Delta K), were visualized using STRUCTURE SELECTOR [23], with ΔK used to identify the optimal K value.

## 3. Results

### 3.1. Genotyping Results and Polymorphism of Microsatellite Loci

Genotyping results are summarized (Table S2), revealing deviations from Hardy–Weinberg equilibrium (HWE) at four loci (HMS07, ASB17, HTG04, HTG07) in Kyrgyz horse, five loci (HMS06, HTG04, HMS03, ASB2, HTG07) in Barkol horse, three loci (HTG04, HMS03, HTG06) in Kazakh horse, four loci (HTG04, HMS03, HTG07, VHL20) in Yili horse, and four loci (HMS07, ASB17, ASB2, HMS2) in Yanqi horse.

Genotyping analysis identified 208 alleles across the 13 microsatellite loci. Breed-specific maximum allele numbers were observed at ASB17 in Kyrgyz, Barkol, Kazakh, and Yanqi horses, while ASB02 exhibited the highest value in Yili horses. Minimum values occurred at: HTG06 in Kyrgyz, Yili, and Yanqi horses; HTG07 in Kazakh horses; and HTG06, ASB23, and HTG07 in Barkol horses. We further assessed the allelic diversity within each race. The results demonstrated that the Barkol horse and Yanqi horse races exhibited the highest number of alleles, each with 147 alleles, followed by the Kyrgyz horse races with 146 alleles. The Kazakh horse races contained 139 alleles, while the Yili horse races showed the lowest allelic count of 137 alleles (Table S2). Polymorphism information content (PIC) ranged from 0.472 (HTG06, Yanqi horse) to 0.924 (ASB17, Barkol horse), with all loci except HTG06 in Yanqi horses (PIC = 0.472) exhibiting high polymorphism (PIC > 0.5).

A total of 43 private alleles were identified across the five races. Yanqi horse race exhibited the highest number of private alleles, totaling 15 (Table S3), with four private alleles having frequencies greater than 5% (HTG04, 139 bp, frequency of 5.6%; HMS03, 155 bp, frequency of 5.0%; AHT04, 144 bp, frequency of 5.0%; ASB23, 165 bp, frequency of 6.7%). The Yili horse breeds exhibited the fewest private alleles with four detected. In the Kyrgyz horse breeds, seven private alleles were detected. The Barkol horse breeds contained nine private alleles, while the Kazakh horse breeds showed eight private alleles including one allele exceeding 5.0% frequency (UCDEQ425, 244bp, frequency of 5.0%); additionally, four private alleles occurred at the UCDEQ425 locus in this breed.

### 3.2. Genetic Diversity Among Five Horse Races in Xinjiang, China

Genetic variation parameters were calculated for the five races (Table 2 and Table S4). Among the five races, the Kyrgyz horse exhibited the lowest values for Ne (6.025), Ho (0.737), and He (0.810). The Barkol horse showed the highest values for Na (11.308, same as Yanqi horse), Ne (6.330), and He (0.816), while the Yili horse had the highest Ho value (0.795) and the lowest Na (10.538).

### 3.3. Genetic Distances and Phylogenetic Relationships Among Five Horse Races in Xinjiang, China

**Figure 1:**
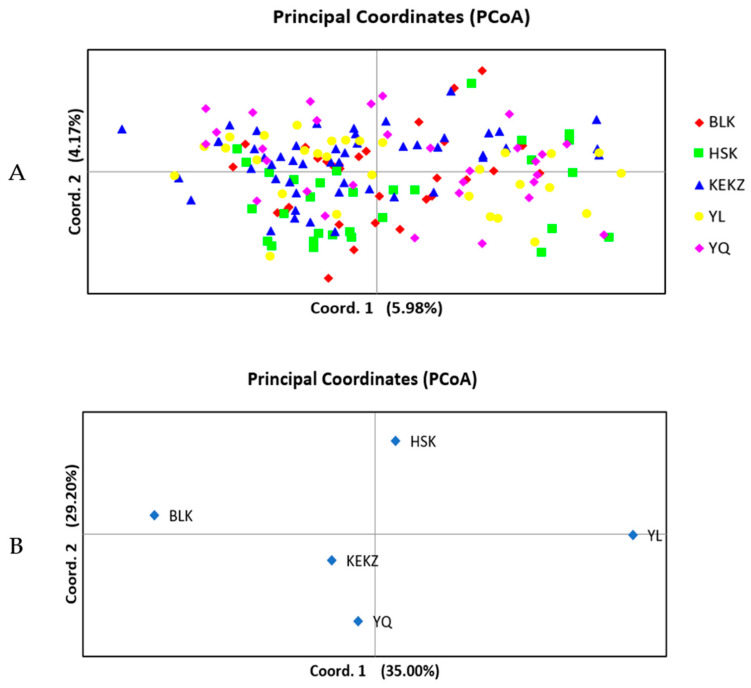
Principal coordinate analysis (PCoA) for five Xinjiang horse races of China. Coordinates 1 and 2 represent the first two principal coordinates. (**A**) Individual PCoA. (**B**) Races PCoA.

**Figure 2:**
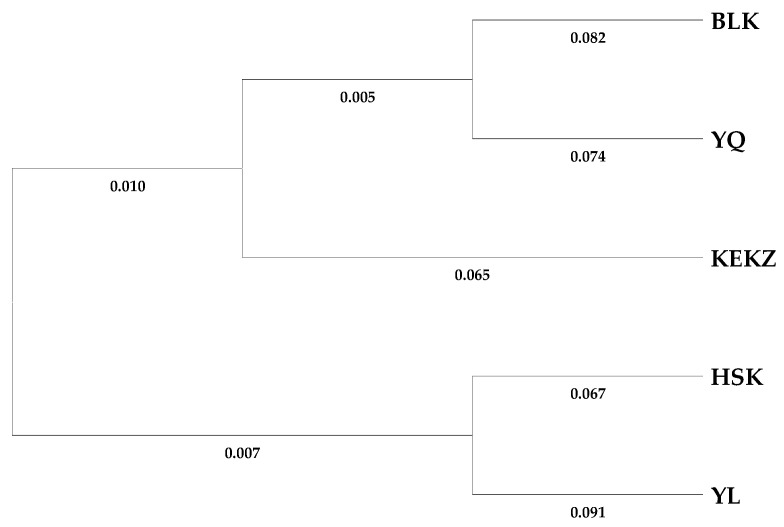
Neighbor-Joining tree showing the genetic distances among the five Xinjiang horse races using Nei’s DA genetic distance.

**Figure 3:**
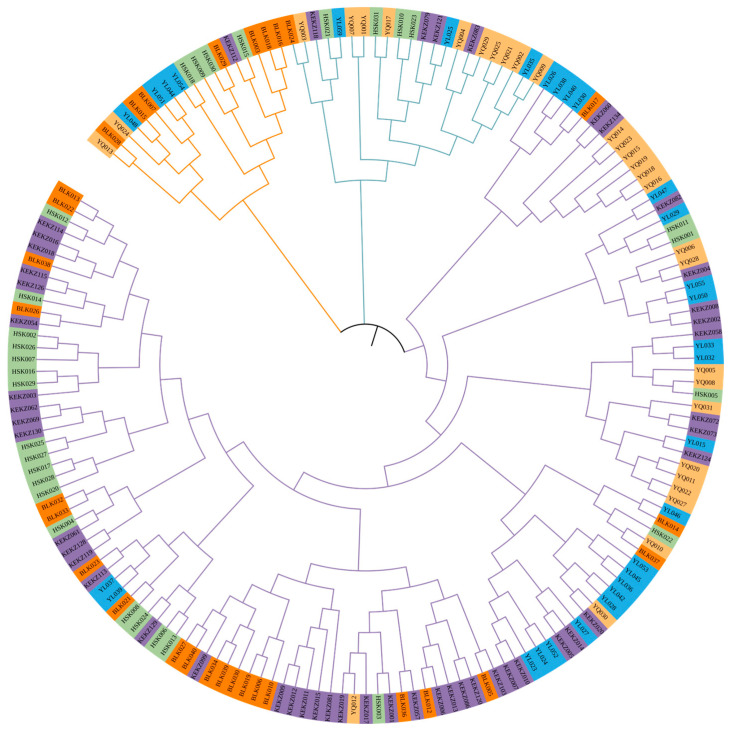
Phylogenetic tree of 170 individuals from the five horse breeds in Xinjiang. Each terminal branch represents an individual horse.

PCoA methods were employed to investigate the potential genetic relationships among different horse races in Xinjiang (Figure 1). In the individual-based analysis, the first three principal coordinates explained 5.98%, 4.17%, and 3.94% of the total variation, respectively, cumulatively accounting for 14.10%. In the group-based analysis, the first three principal coordinates explained 35.00%, 29.20%, and 18.88% of the total variation, respectively, cumulatively accounting for 83.08%. PCoA was also employed to assess the genetic relationships within each of the five horse races (Figure S2). The results revealed considerable genetic distances among individuals within each breed, collectively indicating that the indigenous horse races in Xinjiang maintain a high level of genetic diversity. Phylogenetic analysis grouped Kyrgyz, Yanqi, and Barkol horses into one clade and Kazakh and Yili horses into another distinct clade (Figure 2). The phylogenetic analysis of individuals (Figure 3) reconstructed a tree that clustered the 170 individuals into three distinct clades, comprising 18, 22, and 130 individuals, respectively. The largest clade (Clade III), consisting of 130 individuals, represents the predominant lineage. Notably, all five breeds were distributed across each of the three clades.

Genetic distances among the five horse breeds ranged from 0.125 to 0.219, indicating low differentiation (Table 3). The Kyrgyz and Yanqi horses exhibited the shortest genetic distance (0.125), while the Barkol and Yili horses showed the largest divergence (0.219).

The AMOVA results revealed that the genetic variation occurring among races was 0.08341, while the genetic variation within races was 4.90983, indicating that the majority of genetic variation was concentrated within races, with a low level of genetic differentiation among races (Table 4). The genetic differentiation coefficient (Fst) demonstrated that the overall genetic differentiation among the indigenous horse races in Xinjiang in this study was 2.6% (Table 5). All pairwise Fst values among the five races ranged between 0 and 0.05 (Table 6).

**Figure 4:**
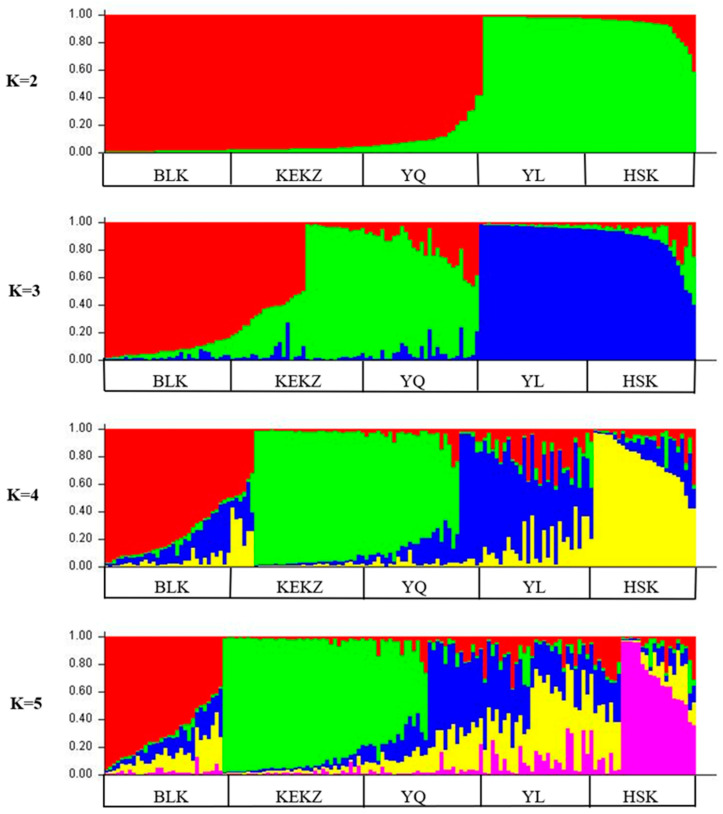
Structure analysis of the five Xinjiang indigenous horse races.

We employed Bayesian clustering to infer race structure and identify the optimal number of genetic clusters (K). The Bayesian analysis evaluated K values ranging from 2 to 6, with race stratification examined for each K value (Figure 4). At K = 2, the Barkol horse, Kyrgyz horse, and Yanqi horse formed one cluster, while the Yili horse and Kazakh horse comprised another distinct cluster. When K = 3, the Barkol horse segregated into a separate cluster, with the Kyrgyz horse and Yanqi horse forming a second cluster, and the Yili horse and Kazakh horse remaining together as a third cluster. The maximum ΔK value occurred at K = 4, defining the optimal cluster number, where the Yili horse and Kazakh horse separated into distinct clusters, the Barkol horse formed an independent cluster, and the Kyrgyz horse and Yanqi horse remained grouped together. At K = 5, the Yili horse race further subdivided into separate clusters while the other races maintained their previous clustering patterns.

## 4. Discussion

This study is the first to analyze the race genetic structure and diversity levels of the Kyrgyz horse and Barkol horse using microsatellite markers and to determine their genetic relationships with three other horse breeds in Xinjiang. The polymorphic information content (PIC) per locus across the five races ranged from 0.472 (HTG06, Yanqi) to 0.924 (ASB17, Barkol). With the exception of locus HTG06 in the Yanqi horse race, all loci were highly polymorphic (PIC > 0.5), indicating their suitability for genetic diversity analysis. This finding aligns with previous studies [8,24], further supporting the utility of microsatellite markers for assessing genetic diversity in local horse breeds, and demonstrates substantial polymorphism within all five races studied.

Among the five races, the Barkol and Yanqi horses exhibited the highest number of alleles, each with 147, indicating a relatively high level of genetic diversity within these groups. In contrast, the Yili horse race showed the lowest allelic count, with only 137 alleles. This reduced allelic diversity may be associated with their breeding strategy, which has extensively incorporated stallions of imported breeds such as Thoroughbred and Arabian horses into local mare races through crossbreeding. It is also noteworthy that the Kyrgyz horse race, despite having a relatively high number of alleles (146), may reflect sampling effects, as this group had a larger number of individuals included in the analysis compared to the other races.

Among the five races analyzed, the Kyrgyz horse exhibited the lowest genetic diversity parameters (Ne = 6.025, Ho = 0.737, He = 0.810), potentially due to its smaller race size. In contrast, the Yili horse displayed the highest observed heterozygosity (Ho = 0.795), indicating maximal heterozygous individuals, potentially reflecting extensive introgression breeding practices. Concurrently, this breed exhibited the lowest number of alleles (Na = 10.538), while the Barkol horse demonstrated the highest genetic diversity (Na = 11.308, Ne = 6.330, He = 0.816) among Xinjiang’s indigenous races. Although the Kyrgyz horse displayed the lowest diversity within these races, its genetic diversity remains relatively high compared to other European and Asian breeds: Terceira pony (Ne = 3.327, Ho = 0.700, He = 0.668) [25], Cleveland Bay horse (Na = 6.200, Ho = 0.465, He = 0.530) [26], Hanoverian Warmblood horse (Na = 6.690, Ho = 0.738, He = 0.734) [7], Misaki horse (Na = 3.000, Ho = 0.509, He = 0.497) [27], Bhutanese horse (Na = 8.060, Ne = 4.960, Ho = 0.790, He = 0.780) [28], Mongolian horse (Na = 8.290, Ho = 0.767, He = 0.752) [29], and Kurdish horse (Na = 7.583, Ne = 4.952, Ho = 0.774, He = 0.783) [30]. Collectively, Xinjiang local horse breeds retain high genetic diversity. However, recent introgression of exotic breeds such as Thoroughbred and Arabian for racing purposes has caused genetic admixture in certain races [31]. This necessitates urgent conservation strategies to safeguard indigenous genetic integrity against exogenous gene flow.

The Na, Ne, Ho, and He values in all five races of this study exceeded those reported for the Guanzhong horse [9]. Compared to other Chinese local breeds such as Jinjiang and Debao horses [8], Xinjiang horses here exhibited higher Na and Ne values and comparable Ho, potentially attributable to Xinjiang’s unique geographic environment and sustained natural selection pressures. Li et al. [11] detected polymorphism at five loci in 51 Kyrgyz horse blood samples using polyacrylamide gel electrophoresis (PAGE), reporting values (Ne = 3.739, Ho = 0.453, PIC = 0.638) substantially lower than ours, likely reflecting limitations in resolution and accuracy associated with the silver-staining methodology. Whereas Wang et al. [10] reported a similar Na value (Na = 11.97) for the Yanqi horse to our findings, significant discrepancies were observed in other parameters (Ne = 7.27, Ho = 0.48, He = 0.70, PIC = 0.67), potentially attributable to their larger sample size and partial marker differences.

In Wright’s F-statistics, the three fixation indices—Fis, Fit, and Fst—serve as indicators for understanding the degree of genetic differentiation among races and the level of inbreeding within races. In real livestock races, the fixation index (F) of subpopulations is not necessarily zero, as the genotype frequencies within each subpopulation may not adhere to the Hardy–Weinberg equilibrium. In this study, the Fis, Fit, and Fst values across all loci for the five races were 0.052 ± 0.029, 0.076 ± 0.029, and 0.026 ± 0.002. The total genetic differentiation among Xinjiang indigenous horse races in this study was 2.6%, reflecting a relatively low level of genetic divergence (0 < Fst < 0.05), which is consistent with some previous studies. For example, the overall differentiation among Chinese local horse breeds was 2.4% [8], the total genetic variation among Chinese domestic buffalo breeds was reported to be 2.8% [32], and the genetic variation among Turkish native donkey races was 1.9% [33]. However, our Fst value is slightly lower than those reported in other studies, such as Leroy et al. [34] found an Fst value of 9.9% in French horse races, Xu et al. [35] reported an Fst value of 6% in Chinese pony races, Yordanov et al. [36] identified an Fst value of 7.8% in Danube horse races, and Wang et al. [37] observed an Fst value of 4.1% in Dezhou donkey races. Collectively, indicating a low overall level of inbreeding among Xinjiang indigenous horse races.

The genetic differentiation among Xinjiang indigenous horse races was investigated using various methods, including PCoA, genetic distance, and Bayesian clustering analysis [22], all of which yielded consistent results. The race-level PCoA indicated that the Kyrgyz horse and Yanqi horse exhibited the closest genetic distance, while the Barkol horse and Yili horse showed the most distant genetic relationship. Phylogenetic tree and Bayesian clustering analysis (K = 2) further supported these findings, grouping the Kyrgyz horse, Yanqi horse, and Barkol horse into one branch, and the Kazakh horse and Yili horse into another branch. This reflects the fundamental genetic differentiation pattern among Xinjiang indigenous horse races. This result may be related to the unique geographical configuration of Xinjiang [38]. It is possible that gene flow occurred between the Barkol horse and the Yanqi horse through the low-elevation hilly areas in eastern Xinjiang. Meanwhile, both the Kyrgyz horse and the Yanqi horse are distributed along the southern foothills of the Tianshan Mountains, where limited geographical isolation may have facilitated relatively frequent genetic exchange between these two races. The maximum value of the likelihood function (ΔK) occurred at K = 4, where the Yili horse and Kazakh horse were separated into distinct clusters, the Barkol horse formed an independent cluster, and the Kyrgyz horse and Yanqi horse remained in the same cluster. This suggests a close genetic relationship between the Kyrgyz horse and Yanqi horse, potentially indicating a shared origin. Xinjiang, a pivotal hub along the ancient Silk Road, has long facilitated extensive genetic exchange among its local horse races. This pattern is clearly reflected in the individual phylogenetic tree, where all five breeds are distributed across three major lineages, supporting their shared genetic background. Further integration of genetic data from horse breeds across Central Asia will be essential to better understand the history and mechanisms of regional genetic exchange.

Our results provide the first characterization of genetic diversity in the Kyrgyz horse and Barkol horse, offering novel insights into the origin of the Kyrgyz horse. These findings extend the genetic resource data for Xinjiang’s indigenous horses and deliver an important scientific basis for their conservation and utilization. This study did not quantify the effective population size (Ne). Future research incorporating multi-year sampling and combining genome-wide SNP markers with pedigree information would allow for more precise estimation of Ne and its dynamics, thereby providing deeper insights into the evolutionary potential and extinction risks of these races.

While this study offers valuable insights into the genetic diversity and relationships of Xinjiang horse breeds, several methodological limitations should be acknowledged. Firstly, although microsatellites are highly polymorphic and remain a standard tool in conservation genetics, they provide limited genomic resolution compared to high-throughput sequencing approaches such as whole-genome sequencing. As a result, they may fail to detect subtle population structures or recent selective events. Secondly, the moderate sample size per breed (*n* = 30), though comparable to those in studies of regional livestock populations, constrains statistical power for accurately estimating rare allele frequencies and fine-scale genetic differentiation. This may also affect the robustness of diversity indices such as expected heterozygosity and allele richness. Future investigations employing larger sample sizes and genome-wide sequencing data will be essential to validate and extend these preliminary findings.

## 5. Conclusions

This study presents the first systematic analysis of the genetic diversity of Kyrgyz and Barkol horses and their relationships with other indigenous horse breeds in Xinjiang, China. The results demonstrate that all five races exhibit high genetic diversity and low inbreeding levels, while revealing a close genetic relationship between Kyrgyz and Yanqi horses that suggests their potential common origin. These findings provide a theoretical basis for the conservation and utilization of Xinjiang’s indigenous horse breeds, particularly offering a genetic foundation for establishing a core conservation race of Kyrgyz horses. We further plan to develop equine dairy products and promote cultural tourism initiatives based on these genetic resources. However, given the limited sample size in this study, future research should incorporate larger sample sizes combined with mitochondrial DNA or whole-genome data for further validation.

## Figures and Tables

**Table 1:** Sampling information of the five horse breeds from Xinjiang.

Breed	Abbreviation	Sample Size	Source Region
Barkol	BLK	30	Barkol County, Xinjiang
Kyrgyz	KEKZ	50	Wuqia County, Xinjiang
Yanqi	YQ	30	Hejing County, Xinjiang
Yili	YL	30	Zhaosu County, Xinjiang
Kazakh	HSK	30	Qinghe County, Xinjiang

**Table 2:** Genetic variability parameters of five horse races in Xinjiang, China.

Pop	Sample Size	Na	Ne	Ho	He
BLK	30	11.308	6.330	0.792	0.816
KEKZ	50	11.231	6.025	0.737	0.810
YQ	30	11.308	6.326	0.753	0.814
YL	30	10.538	6.207	0.795	0.815
HSK	30	10.692	6.305	0.782	0.820
Mean ± SE		11.015 ± 0.371	6.239 ± 0.129	0.772 ± 0.256	0.815 ± 0.004

Na: No. Alleles, Ne: No. Effective Alleles, Ho: observed heterozygosity, and He: expected heterozygosity.

**Table 3:** Pairwise matrix of Nei genetic distance of five horse races in Xinjiang, China.

Races	BLK	KEKZ	YQ	YL	HSK
BLK	0.000				
KEKZ	0.128	0.000			
YQ	0.156	0.125	0.000		
YL	0.219	0.153	0.164	0.000	
HSK	0.149	0.148	0.181	0.157	0.000

**Table 4:** Analysis of molecular variance for five genetic groups of Xinjiang indigenous horse based on allele frequencies from 13 microsatellite markers.

Source of Variation	d.f	Sum of Squares	Variance Component
Among populations	4	39.657	0.08341
Within populations	295	1448.400	4.90983
Total	299	1488.057	4.99324

d.f. degrees of freedom.

**Table 5:** F-Statistics over All Pops for each Locus.

Pop	Locus	Fis	Fit	Fst
All pops	HMS07	0.103	0.118	0.017
	ASB17	0.052	0.075	0.024
	HMS06	0.050	0.079	0.031
	HTG04	0.260	0.281	0.028
	HMS03	0.117	0.136	0.022
	HTG06	−0.053	−0.023	0.028
	AHT04	−0.135	−0.121	0.012
	ASB23	0.064	0.102	0.041
	ASB2	0.032	0.052	0.020
	HMS2	0.007	0.031	0.023
	HTG07	0.199	0.231	0.040
	UCDEQ425	−0.052	−0.023	0.028
	VHL20	0.031	0.053	0.022
	Mean ± SE	0.052 ±0.029	0.076 ±0.029	0.026 ±0.002

Fis deviation from Hardy–Weinberg proportions (within-population inbreeding coefficient); Fit inbreeding coefficient of an individual relative into the whole population; Fst coefficient of differentiation, fixation index.

**Table 6:** Percentage of estimated genetic distance from the Fst Values for Xinjiang indigenous horse from the five races.

Races	BLK	KEKZ	YQ	YL	HSK
BLK	0.000				
KEKZ	0.014	0.000			
YQ	0.016	0.013	0.000		
YL	0.022	0.016	0.017	0.000	
HSK	0.016	0.016	0.019	0.016	0.000

## Data Availability

All data supporting the findings of this study are available within the article.

## Supplementary Materials

The following supporting information can be downloaded at: https://www.mdpi.com/article/10.3390/ani15182690/s1, Table S1: List of microsatellite motifs and their primer sequences; Table S2: Summary of genotyping results; Table S3: Summary of Private Alleles by Population; Table S4: Genetic variability parameters; Table S5: Analysis of 10 independent runs of STRUCTURE with STRUCTURESELECTOR to obtain the mean of the posterior probability ln P(G|K) (Ln P(K)) with its standard deviation (Stdev Ln P(K)), Ln’(K), |Ln’’(K)| and ΔK (Delta K) for K = 2–6. The maximum of the mean Ln P(K) and ΔK are reached at K = 4 with this row highlighted in yellow in the results table.; Figure S1: Photographs of the five horse breeds from Xinjiang.; Figure S2: Individual Principal Coordinates Analysis (PCoA) plots for the five breeds.; Figure S3: Plot of the mean Ln P(K) with the corresponding standard deviation (Stdev) from 10 repetitions of the STRUCTURE runs for K = 2 to 6. The maximum of Ln P(K) is indicated by vertical red dashed lines and the standard deviations for each K by gray vertical lines.; Figure S4: A line graph of the ΔK (Delta K) values obtained from 10 repeated runs of STRUCTURE when the K value ranges from 2 to 6. The maximum value of ΔK (Delta K) is marked by a vertical red dashed line.
