# Supplementary material for: Analysis of Genetic Diversity and Race Genetic Structure of Major Horse Breeds in Xinjiang, China
Source: Animals (Basel). 2025 Sep 14;15(18):2690. doi: 10.3390/ani15182690 (PMC12466770; doi:10.3390/ani15182690)
Supplement: Supplementary file 1 [file animals-15-02690-s001.zip › animals-3838417-supplementary.pdf]

Table S1. List of microsatellite motifs and their primer sequences

Loci	Primer sequences (5'-3')	Tm（℃)	Fragment size (bp)
HTG04F	CTATCTCAGTCTTCATTGCAGGAC	54	128-144
HTG04R	CTCCCTCCCTCCCTCTGTTCTC		
ASB17F	GAGGGCGGTACCTTTGTACC	55	88-128
ASB17R	ACCAGTCAGGATCTCCACCG		
HMS06F	GAAGCTGCCAGTATTCAACCATTG	54	156-172
HMS06R	CTCCATCTTGTGAAGTGTAACTCA		
HTG06F	CCTGCTTGGAGGCTGTGATAAGAT	54	81-103
HTG06R	GTTCACTGAATGTCAAATTCTGCT		
ASB23F	GAGGTTTGTAATTGGAATG	55	145-175
ASB23R	GAGAAGTCATTTTTAACACCT		
HMS07F	CAGGAAACTCATGTTGATACCATC	55	170-180
HMS07R	TGTTGTTGAAACATACCTGACTGT		
AHT04F	AACCGCCTGAGCAAGGAAGT	58.6	133-165
AHT04R	GCTCCCAGAGAGTTTACCCT		
HMS3F	CCATCCTCACTTTTTCACTTTGTT	55	144-172
HMS3R	CCAACTCTTTGTCACATAACAAGA		
VHL20F	CAAGTCCTCTTACTTGAAGACTAG	58.6	89-112
VHL20R	AACTCAGGGAGAATCTTCCTCAG		
HMS2F	ACGGTGGCAACTGCCAAGGAAG	53	213-239
HMS2R	CTTGCAGTCGAATGTGTATTAAATG		
ASB2F	CCTTCCGTAGTTTAAGCTTCTG	53.6	161-189
ASB2R	CACAACTGAGTTCTCTGATAGG		
HTG07F	CCTGAAGCAGAACATCCCTCCTTG	55	118-138
HTG07R	ATAAAGTGTCTGGGCAGAGCTGCT		
UCDEQ425F	AGCTGCCTCGTTAATTCA	54	228-248
UCDEQ425R	CTCATGTCCGCTTGTCTC		

Table S2. Summary of genotyping results

Pop	Loci	HMS07	ASB17	HMS06	HTG04	HMS03	HTG06	AHT04	ASB23	ASB2	HMS2	HTG07	UCDEQ425	VHL20
KEKZ	K	14	22	8	11	12	6	10	11	17	5	10	9	11
	N	50	50	50	50	50	50	50	49	50	50	48	50	50
	PIC	0.845	0.891	0.828	0.761	0.827	0.534	0.825	0.835	0.887	0.728	0.726	0.789	0.780
	HWE	*	*	ns	***	ns	ns	ns	ns	ns	ns	***	ns	ns
	NPA	2	0	0	0	0	0	0	1	0	0	1	0	0
BLK	K	14	23	9	11	12	8	9	8	16	10	8	10	9
	N	30	30	30	30	30	30	30	30	30	30	30	30	30
	PIC	0.852	0.924	0.771	0.833	0.832	0.588	0.823	0.781	0.879	0.825	0.727	0.734	0.781
	HWE	ns	ns	**	***	**	ns	ns	ns	*	ns	***	ns	ns
	NPA	0	1	1	0	1	2	0	1	0	1	2	1	0
HSK	K	12	25	7	7	11	6	13	8	15	8	5	12	10
	N	30	30	30	30	30	30	30	30	29	30	29	30	28
	PIC	0.781	0.909	0.909	0.635	0.840	0.724	0.815	0.816	0.896	0.745	0.724	0.793	0.846
	HWE	ns	ns	ns	***	*	*	ns	ns	ns	ns	ns	ns	ns
	NPA	0	1	0	0	0	0	2	1	0	0	0	4	1
YL	K	13	16	8	11	10	6	10	9	17	9	7	9	12
	N	30	30	30	30	30	30	30	30	29	30	30	30	30
	PIC	0.872	0.883	0.789	0.766	0.844	0.637	0.828	0.748	0.904	0.740	0.761	0.678	0.859
	HWE	ns	ns	ns	***	**	ns	ns	ns	ns	ns	*	ns	*
	NPA	0	0	1	1	1	0	0	0	0	0	0	0	1
YQ	K	12	24	8	12	14	4	11	11	16	7	8	9	11
	N	30	30	30	27	30	30	30	30	29	30	27	30	24
	PIC	0.794	0.912	0.812	0.778	0.843	0.472	0.832	0.829	0.894	0.763	0.820	0.777	0.792
	HWE	***	***	ns	ns	ns	ns	ns	ns	**	*	ns	ns	ns
	NPA	1	3	1	1	3	0	2	2	0	0	0	0	2

Notes: K: the number of alleles, N: the number of individuals at the locus. PIC: polymorphic information content, PIC < 0.25, low polymorphism; 0.25 < PIC < 0.5, intermediate polymorphism; PIC > 0.5, high polymorphism. HWE: Hardy–Weinberg Equilibrium, ns = not significant,*P < 0.05, **P < 0.01, ***P < 0.001. NPA: number of private alleles. KEKZ, Kyrgyz horse; BLK, Barkol horse; HSK, Kazakh horse; YL, Yili horse; YQ, Yanqi horse.

Table S3. Summary of Private Alleles by Population

Pop	Locus	Allele	Freq
BLK	ASB17	133	0.017
BLK	HMS06	171	0.017
BLK	HMS03	159	0.017
BLK	HTG06	99	0.017
BLK	HTG06	102	0.033
BLK	ASB23	150	0.033
BLK	HMS2	216	0.017
BLK	HTG07	119	0.017
BLK	UCDEQ425	246	0.033
HSK	ASB17	118	0.017
HSK	AHT04	130	0.017
HSK	AHT04	136	0.017
HSK	UCDEQ425	236	0.017
HSK	UCDEQ425	240	0.017
HSK	UCDEQ425	242	0.017
HSK	UCDEQ425	244	0.050
HSK	VHL20	107	0.018
KEKZ	HMS07	161	0.010
KEKZ	HMS07	170	0.020
KEKZ	ASB17	143	0.010
KEKZ	ASB23	168	0.041
KEKZ	ASB2	172	0.020
KEKZ	HTG07	121	0.021
KEKZ	VHL20	91	0.020
YL	HMS06	151	0.017
YL	HTG04	134	0.017
YL	HMS03	157	0.017
YL	VHL20	93	0.017
YQ	HMS07	183	0.017
YQ	ASB17	87	0.017
YQ	ASB17	95	0.017
YQ	ASB17	120	0.017
YQ	HMS06	159	0.017
YQ	HTG04	139	0.056
YQ	HMS03	154	0.017
YQ	HMS03	155	0.050
YQ	HMS03	168	0.017
YQ	AHT04	144	0.050
YQ	AHT04	146	0.017
YQ	ASB23	165	0.067
YQ	ASB23	176	0.033
YQ	VHL20	99	0.021

YQ	VHL20	103	0.021

Table S4. Genetic variability parameters

Pop	Locus	N	Na	Ne	I	Ho	He	uHe	F
BLK	HMS07	30	14.000	7.438	2.260	0.867	0.866	0.880	-0.001
	ASB17	30	23.000	13.846	2.872	0.867	0.928	0.944	0.066
	HMS06	30	9.000	4.852	1.832	0.633	0.794	0.807	0.202
	HTG04	30	11.000	6.642	2.095	0.667	0.849	0.864	0.215
	HMS03	30	12.000	6.618	2.103	0.900	0.849	0.863	-0.060
	HTG06	30	8.000	2.616	1.350	0.667	0.618	0.628	-0.079
	AHT04	30	9.000	6.294	1.970	0.967	0.841	0.855	-0.149
	ASB23	30	8.000	5.143	1.819	0.800	0.806	0.819	0.007
	ASB2	30	16.000	8.955	2.439	0.867	0.888	0.903	0.024
	HMS2	30	10.000	6.406	1.989	0.900	0.844	0.858	-0.066
	HTG07	30	8.000	4.128	1.661	0.567	0.758	0.771	0.252
	UCDEQ425	30	10.000	4.206	1.770	0.833	0.762	0.775	-0.093
	VHL20	30	9.000	5.143	1.862	0.767	0.806	0.819	0.048
	Mean		11.308	6.330	2.002	0.792	0.816	0.830	0.028
HSK	HMS07	30	12.000	7.531	2.199	0.800	0.867	0.882	0.078
	ASB17	30	25.000	11.688	2.815	0.933	0.914	0.930	-0.021
	HMS06	30	7.000	4.826	1.701	0.900	0.793	0.806	-0.135
	HTG04	30	7.000	3.109	1.410	0.467	0.678	0.690	0.312
	HMS03	30	11.000	6.923	2.131	0.633	0.856	0.870	0.260
	HTG06	30	6.000	4.138	1.574	0.767	0.758	0.771	-0.011
	AHT04	30	13.000	6.000	2.084	0.900	0.833	0.847	-0.080
	ASB23	30	8.000	6.122	1.896	0.833	0.837	0.851	0.004
	ASB2	29	15.000	10.383	2.501	0.966	0.904	0.920	-0.068
	HMS2	30	8.000	4.500	1.692	0.800	0.778	0.791	-0.029
	HTG07	29	5.000	4.195	1.519	0.621	0.762	0.775	0.185
	UCDEQ425	30	12.000	5.357	1.985	0.800	0.813	0.827	0.016
	VHL20	28	10.000	7.193	2.112	0.750	0.861	0.877	0.129
	Mean		10.692	6.305	1.971	0.782	0.820	0.834	0.049
KEKZ	HMS07	50	14.000	7.112	2.225	0.680	0.859	0.868	0.209
	ASB17	50	22.000	9.901	2.630	0.780	0.899	0.908	0.132
	HMS06	50	8.000	6.545	1.943	0.780	0.847	0.856	0.079
	HTG04	50	11.000	4.647	1.861	0.480	0.785	0.793	0.388
	HMS03	50	12.000	6.443	2.100	0.700	0.845	0.853	0.171
	HTG06	50	6.000	2.300	1.176	0.680	0.565	0.571	-0.203
	AHT04	50	10.000	6.386	2.029	0.980	0.843	0.852	-0.162
	ASB23	50	11.000	6.754	2.071	0.755	0.852	0.861	0.114
	ASB2	50	17.000	9.524	2.497	0.800	0.895	0.904	0.106
	HMS2	50	5.000	4.274	1.522	0.820	0.766	0.774	-0.070
	HTG07	50	10.000	4.129	1.713	0.542	0.758	0.766	0.285
	UCDEQ425	50	9.000	5.330	1.862	0.840	0.812	0.821	-0.034
	VHL20	50	11.000	4.980	1.942	0.740	0.799	0.807	0.074
	Mean		11.231	6.025	1.967	0.737	0.810	0.818	0.084

YL	HMS07	30	13.000	8.571	2.312	0.800	0.883	0.898	0.094
	ASB17	30	16.000	9.184	2.469	0.900	0.891	0.906	-0.010
	HMS06	30	8.000	5.389	1.810	0.733	0.814	0.828	0.100
	HTG04	30	11.000	4.800	1.881	0.533	0.792	0.805	0.326
	HMS03	30	10.000	7.115	2.079	0.833	0.859	0.874	0.030
	HTG06	30	6.000	3.169	1.344	0.733	0.684	0.696	-0.071
	AHT04	30	10.000	6.498	2.034	1.000	0.846	0.860	-0.182
	ASB23	30	9.000	4.478	1.744	0.700	0.777	0.790	0.099
	ASB2	29	17.000	11.139	2.609	0.897	0.910	0.926	0.015
	HMS2	30	9.000	4.337	1.714	0.700	0.769	0.782	0.090
	HTG07	30	7.000	4.749	1.718	0.733	0.789	0.803	0.071
	UCDEQ425	30	9.000	3.468	1.565	0.800	0.712	0.724	-0.124
	VHL20	30	12.000	7.792	2.231	0.967	0.872	0.886	-0.109
	Mean		10.538	6.207	1.962	0.795	0.815	0.829	0.025
YQ	HMS07	30	12.000	5.373	1.989	0.700	0.814	0.828	0.140
	ASB17	30	24.000	12.162	2.802	0.833	0.918	0.933	0.092
	HMS06	30	8.000	6.020	1.881	0.833	0.834	0.848	0.001
	HTG04	27	12.000	4.959	1.982	0.741	0.798	0.813	0.072
	HMS03	30	14.000	7.031	2.218	0.700	0.858	0.872	0.184
	HTG06	30	4.000	2.088	0.944	0.467	0.521	0.530	0.104
	AHT04	30	11.000	6.593	2.096	0.933	0.848	0.863	-0.100
	ASB23	30	11.000	6.498	2.068	0.767	0.846	0.860	0.094
	ASB2	29	16.000	10.194	2.512	0.828	0.902	0.918	0.082
	HMS2	30	7.000	4.813	1.709	0.700	0.792	0.806	0.116
	HTG07	27	8.000	6.231	1.944	0.667	0.840	0.855	0.206
	UCDEQ425	30	9.000	5.056	1.849	0.833	0.802	0.816	-0.039
	VHL20	24	11.000	5.213	1.998	0.792	0.808	0.825	0.020
	Mean		11.308	6.326	1.999	0.753	0.814	0.828	0.075

Table S5. Analysis of 10 independent runs of STRUCTURE with STRUCTURESELECTOR to obtain the mean of the posterior probability ln P(G|K) (Ln P(K)) with its standard deviation (Stdev Ln P(K)), Ln′(K), |Ln′′(K)| and ΔK (Delta K) for K = 2-6. The maximum of the mean Ln P(K) and ΔK are reached at K=4 with this row highlighted in yellow in the results table.

K	Reps	Mean LnP(K)	Stdev LnP(K)	Ln'(K)	|ln″(K)|	Delta K
2	10	-9249.13000	5.57974	NA	NA	NA
3	10	-9187.05000	56.68404	62.08000	54.84000	0.96747
4	10	-9179.81000	23.32573	7.24000	289.72000	12.42062
5	10	-9462.29000	85.55538	-282.48000	190.42000	2.22569
6	10	-9554.35000	153.71661	-92.06000	NA	NA

NA: not applicable.

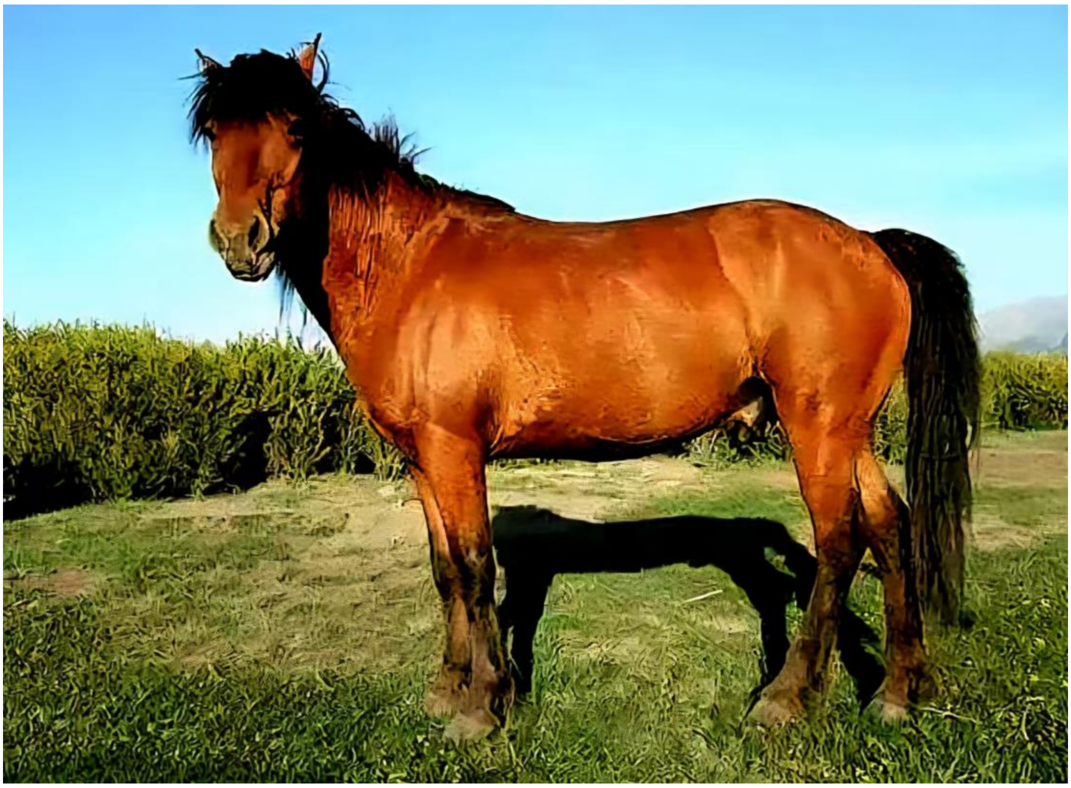

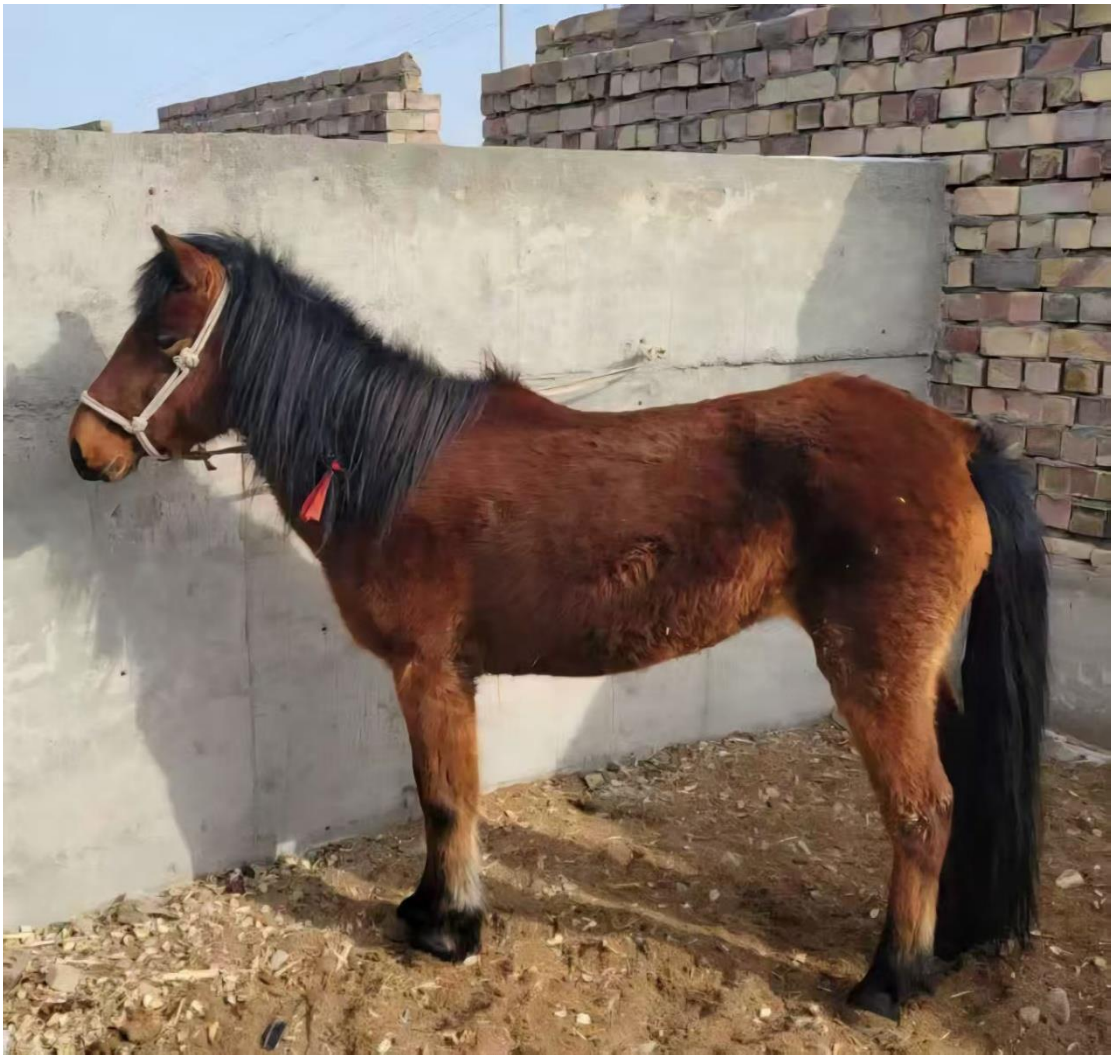

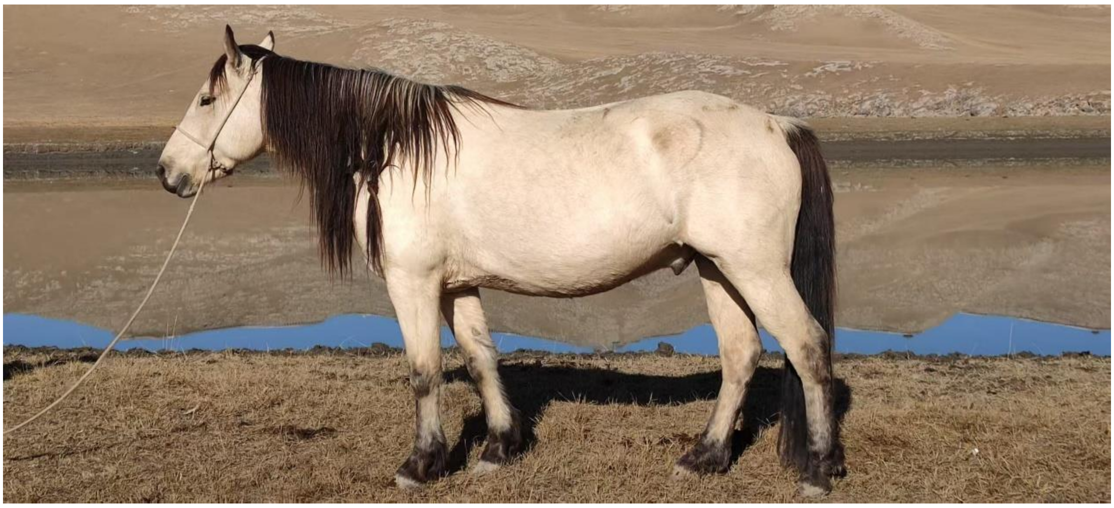

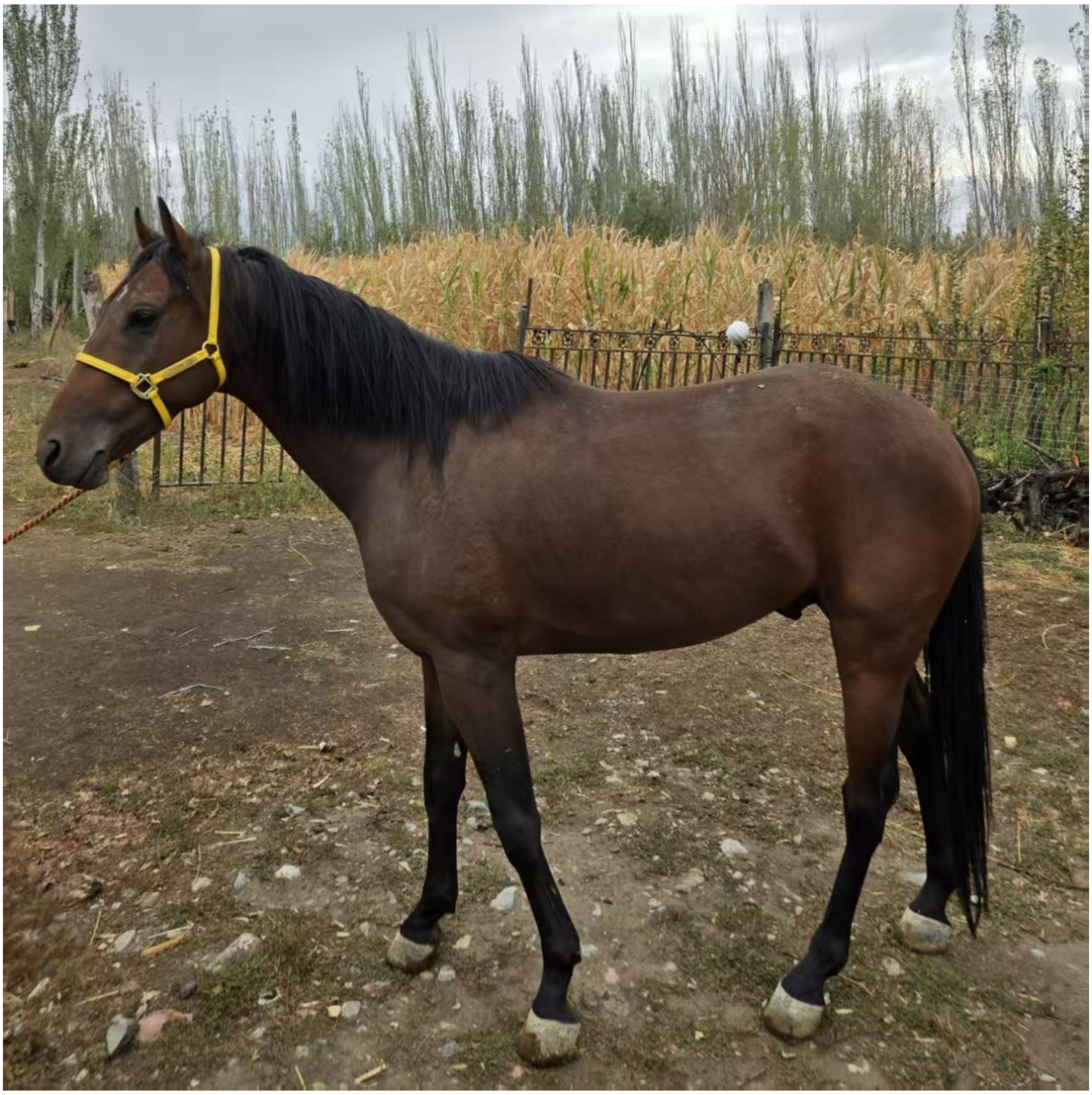

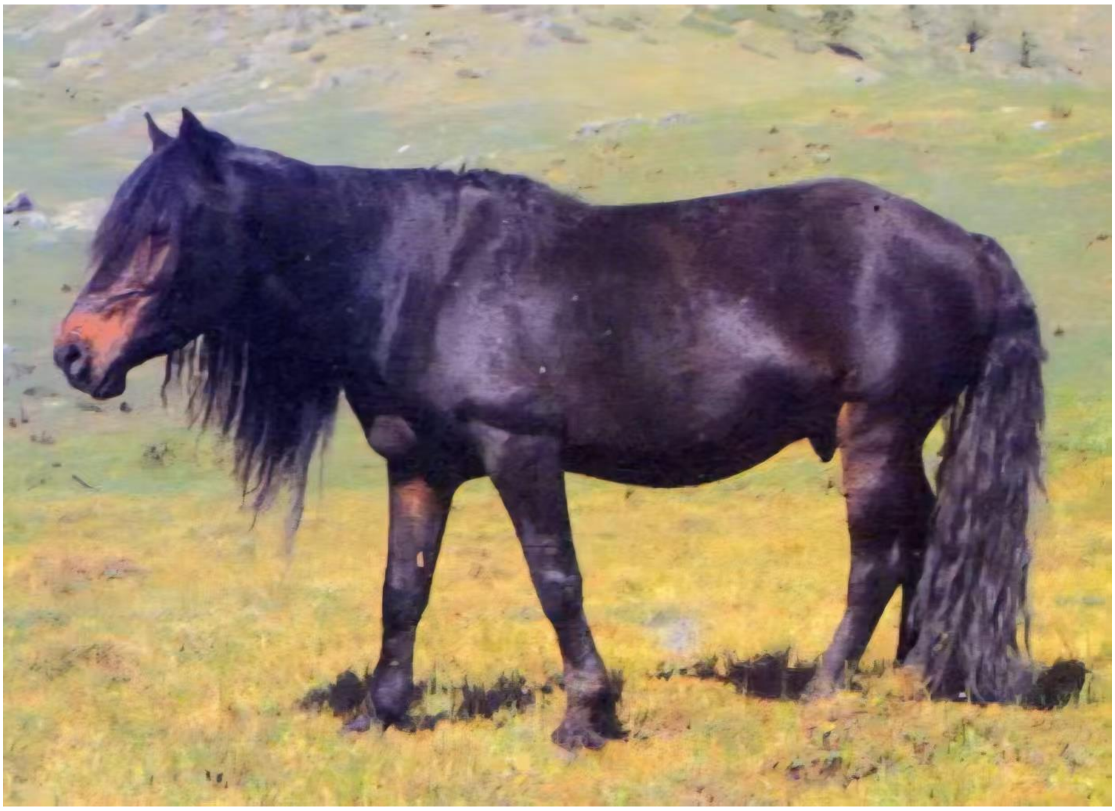

Figure S1. Photographs of the five horse breeds from Xinjiang .
They are presented in the following order: BLK, Barkol horse; KEKZ, Kyrgyz horse; YQ, Yanqi horse; YL, Yili horse; HSK, Kazakh horse.

Principal Coordinates (PCoA) of the BLK horse

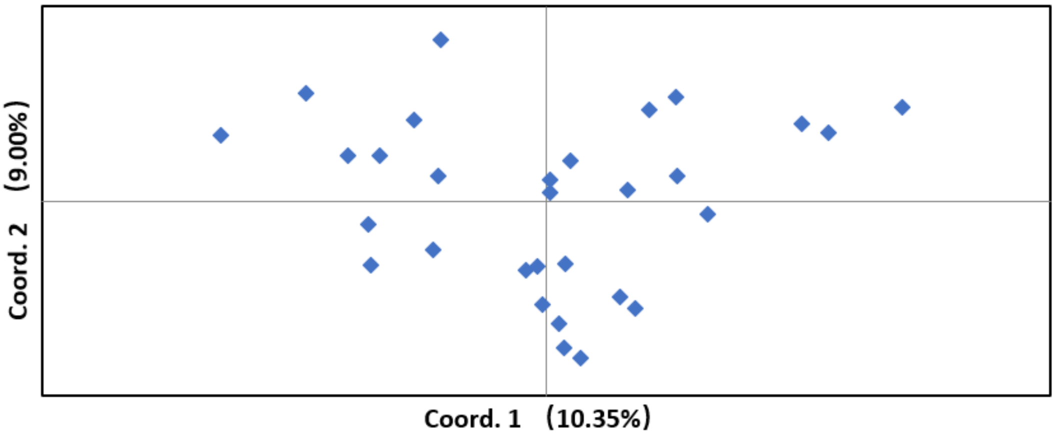

Principal Coordinates (PCoA) of the KEKZ horse

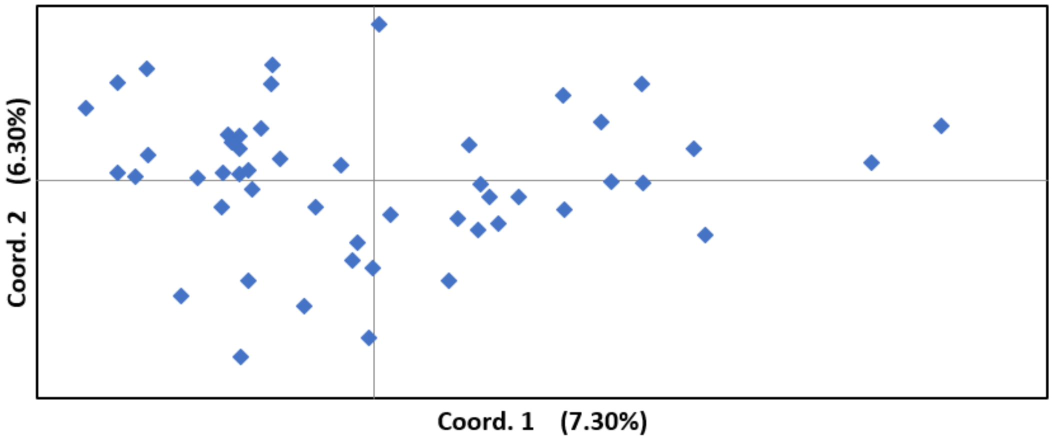

Principal Coordinates (PCoA) of the YQ horse

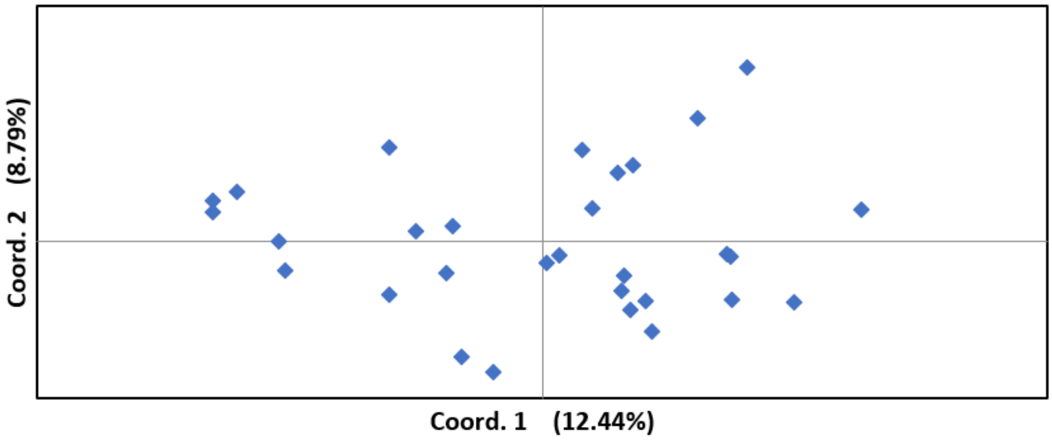

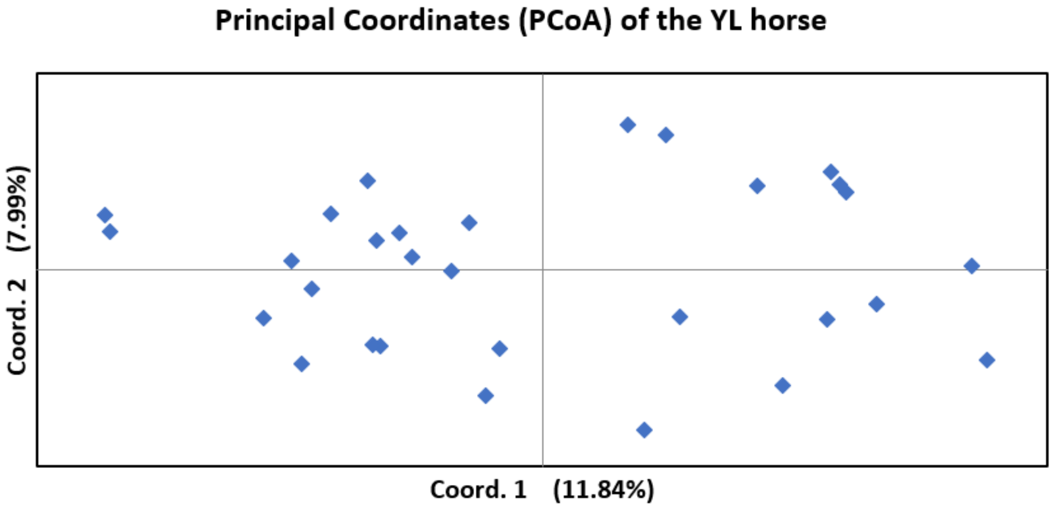

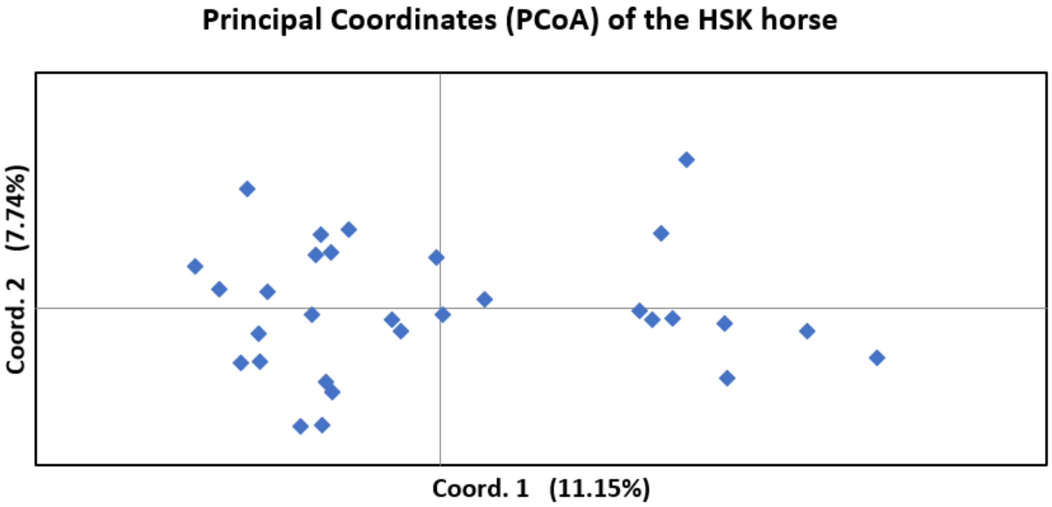

Figure S2. Individual Principal Coordinates Analysis (PCoA) plots for the five breeds.

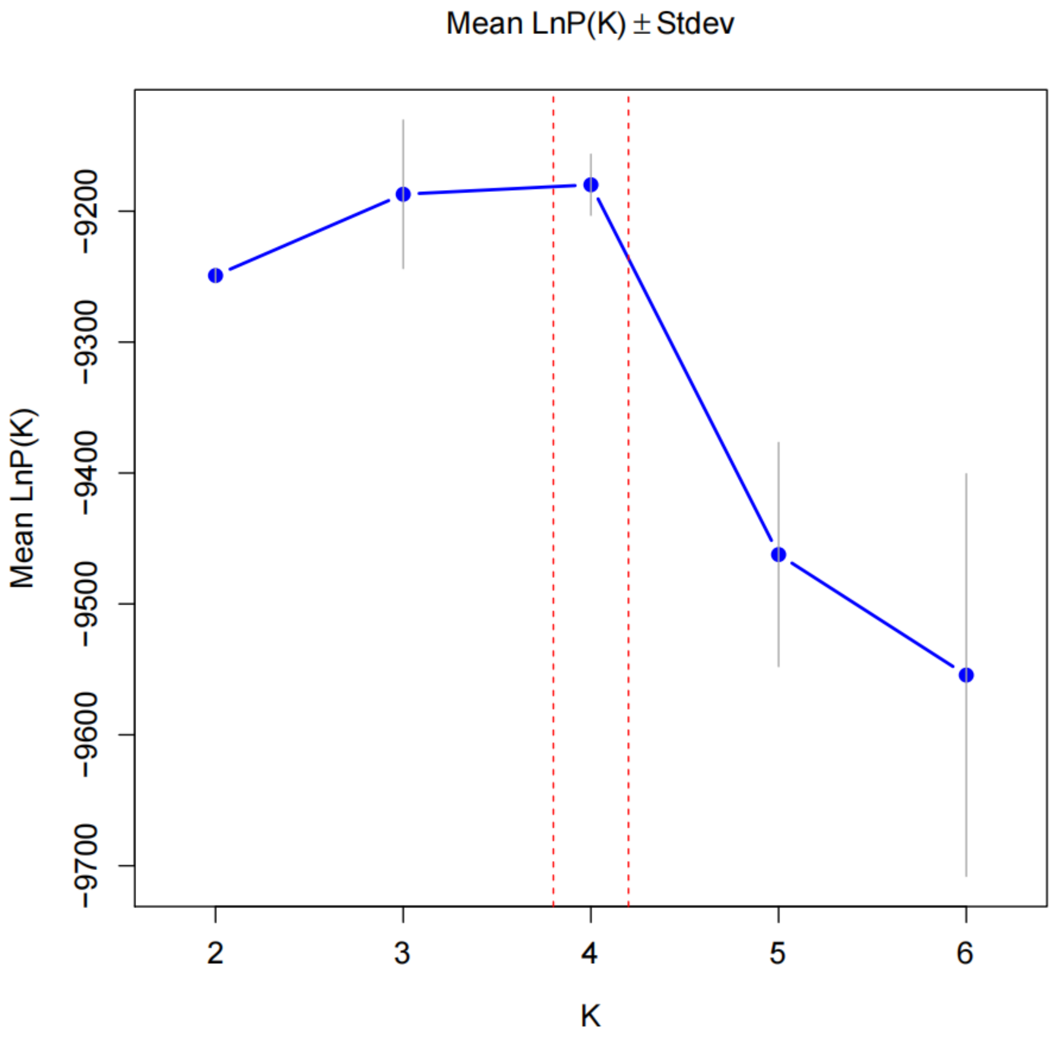

Figure S3. Plot of the mean Ln P(K) with the corresponding standard deviation (Stdev) from 10 repetitions of the STRUCTURE runs for K = 2 to 6. The maximum of Ln P(K) is indicated by vertical red dashed lines and the standard deviations for each K by gray vertical lines.

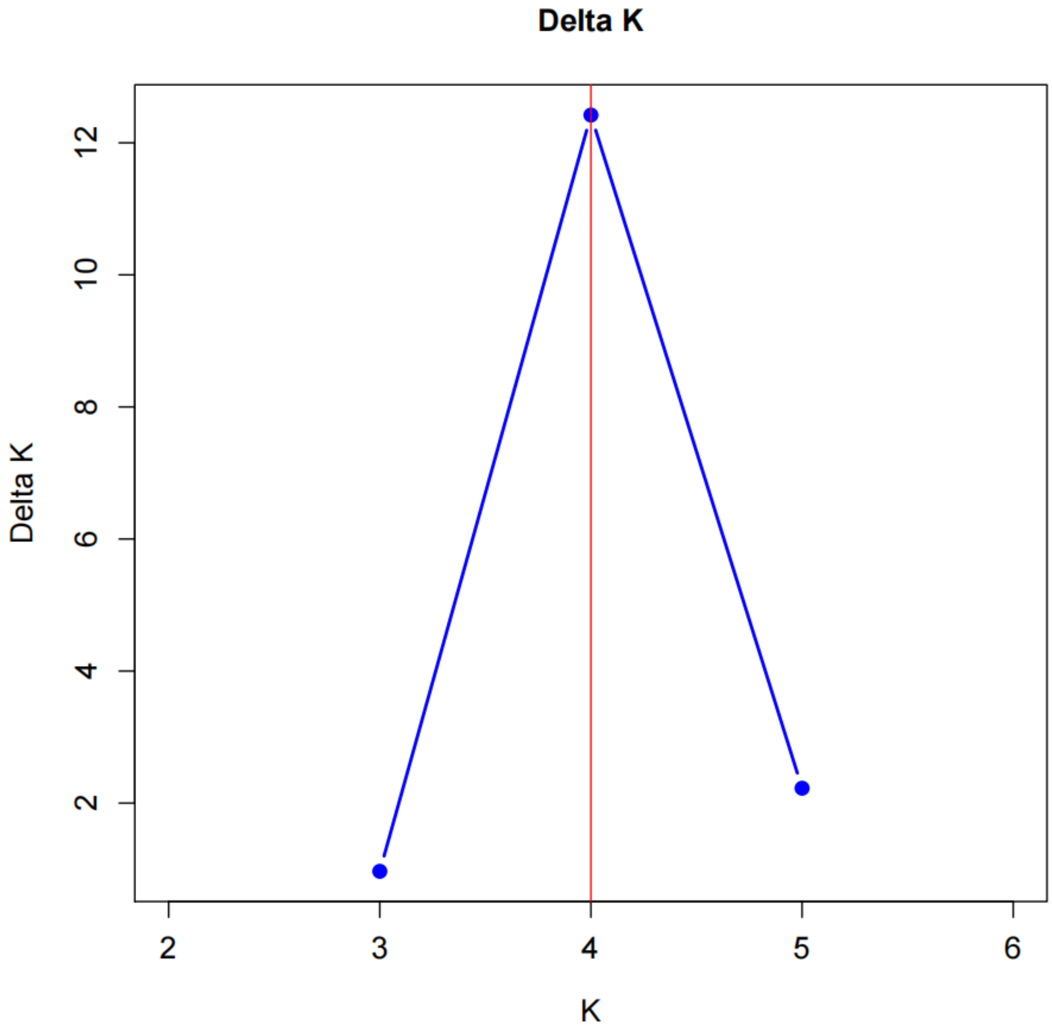

Figure S4. A line graph of the ΔK (Delta K) values obtained from 10 repeated runs of STRUCTURE when the K value ranges from 2 to 6. The maximum value of ΔK (Delta K) is marked by a vertical red dashed line.
